# A Novel Information Retrieval Model for High-Throughput Molecular Medicine Modalities

**DOI:** 10.4137/cin.s964

**Published:** 2009-02-09

**Authors:** Firas H. Wehbe, Steven H. Brown, Pierre P. Massion, Cynthia S. Gadd, Daniel R. Masys, Constantin F. Aliferis

**Affiliations:** 1 Department of Biomedical Informatics, Vanderbilt University, Nashville, TN, U.S.A; 2 U.S. Department of Veteran Affairs, U.S.A; 3 Division of Allergy, Pulmonary, and Critical Care Medicine, Department of Medicine, Vanderbilt University, Nashville, TN, U.S.A; 4 Center of Health Informatics and Bioinformatics, New York University; 5 Department of Pathology, New York University School of Medicine

**Keywords:** information retrieval, molecular medicine, semantic model, clinical bioinformatics, predictive computational models

## Abstract

Significant research has been devoted to predicting diagnosis, prognosis, and response to treatment using high-throughput assays. Rapid translation into clinical results hinges upon efficient access to up-to-date and high-quality molecular medicine modalities.

We first explain why this goal is inadequately supported by existing databases and portals and then introduce a novel semantic indexing and information retrieval model for clinical bioinformatics. The formalism provides the means for indexing a variety of relevant objects (e.g. papers, algorithms, signatures, datasets) and includes a model of the research processes that creates and validates these objects in order to support their systematic presentation once retrieved.

We test the applicability of the model by constructing proof-of-concept encodings and visual presentations of evidence and modalities in molecular profiling and prognosis of: (a) diffuse large B-cell lymphoma (DLBCL) and (b) breast cancer.

## Introduction

The goal of Molecular Medicine is to diagnose and find treatments for human diseases by the application of tools of molecular and cell biology (Sobie et al. 2003). In recent years, researchers have begun to link tissue molecular profiles—such as gene expression information—of individual patients to relevant disease outcomes such as diagnosis (Quackenbush, 2006), prognosis (Ntzani and Ioannidis, 2003), and response to treatment (Ross and Ginsburg, 2003). Knowledge discovered from large-scale genomic and molecular biology data is already being put to clinical use (van’t Veer et al. 2002) and several clinical studies are in the development or validation phase (Simon, 2005).

The field of pharmocogenomics, for example, applies whole genome analysis technologies to predict drug treatment response and adverse drug reaction susceptibility based on individual genetic variability (Marsh and McLeod, 2006; Ross et al. 2004). For instance, an inherited genetic trait places some individuals at risk for adverse drug reactions (diarrhea, neutropenia) to the antineoplastic drug irinotecan (Ando et al. 2000; Ciotti et al. 1998; Innocenti et al. 2004). Individuals with the most common variant allele (UGT1A1*28) have lower expression levels of an enzyme that deactivates irinotecan. The FDA requires that the related genotype and dosing guideline information be included in the irinotecan package insert (Food and Drug Administration 2008). Other mutations are associated with a good clinical prognosis (Bell et al. 2005) and positive response to certain classes of drugs (Lynch et al. 2004). A listing of drug-related genomic biomarkers is available on the FDA website (Food and Drug Administration, 2008).

In a typical scenario, a molecular assay is performed on tissue obtained from a patient. Then, a decision model computes, based on the assay results, the “predicted” clinical outcome of the patient’s disease. For example, the U.S. Food and Drug Administration approved in February of 2007 the first high-dimensional molecular test to predict the recurrence of breast cancer within five to ten years. Many similar tests are expected to follow (Couzin, 2007).

Discovering clinically significant knowledge from large-scale genome and molecular biology information is a complicated scientific process that draws from multiple overlapping sources of data describing complex interactions at the genomic, proteomic, or other “omic” levels. High throughput “omic” experimental methods generate data that can have hundreds or even hundreds of thousands of data-points per sample. Such data are difficult to process manually and require sophisticated computation. Decision models that process the resulting data are also complex and draw from a variety of disciplines including biostatistics and machine learning. Furthermore, there is great variability in the methods that evaluate these predictive models’ validity, generalizability, and supporting evidence (Simon, 2005).

For advances in molecular medicine to come to clinical fruition, it is crucial for clinical and translational researchers to have access to relevant, up-to-date, and correct information about known molecular medicine modalities (Mathew et al. 2007), such as research datasets, research methods, known and validated decision models, and related evidence. Therefore the important problem of retrieving and organizing the vast amount of information issued from molecular medicine research needs to be addressed. The inherent complexity of this domain and the fast pace of scientific discovery make this problem particularly challenging.

## Problem Statement

Our goal is to develop a general purpose information retrieval system that satisfies the following two requirements:

1. The system should be able to index, retrieve and organize most methods of molecular profiling, most forms of predictive computational models, many types of clinical outcome, as well as supporting evidence and computational resources.
2. The knowledgebase needs to be comprehensive and up to date. This requires simple, cheap, fast, and scalable methods to build the knowledge base and to keep it current. To keep up with the rapid pace of discovery in clinical bioinformatics, these methods have to be automated or semiautomated in the worst case.

For this system to support the first requirement, its underlying knowledge representation formalism has to convey the semantic complexity of the clinical bioinformatics domain; on the other hand, the underlying formalism has to be simple enough to support the second requirement of relying on scalable automated methods. The problem, therefore, is to develop a framework and semantic model that balance these two requirements.

This system will also have to accommodate a wide range of query types. Consider the following query examples to be posed by clinicians and/or clinical and translational researchers:

- **Example Query 1:** *“What models exist that predict the response to the chemotherapy regiment (CHOP) in patients with diffuse large B-cell lymphoma (DLBCL)?”* In this query, the following entities are specified: “disease” is specified as “DLBCL”; “clinical outcome” is specified as “response to CHOP”. Notice that this question leaves the specific method of “molecular profiling” open. This query might be posed by an oncologist looking for up-to-date knowledge to guide her choice of treatment strategy for her DLBCL patient.
- **Example Query 2:** *“What models exist that predict response to the chemotherapy regiment (CHOP) based on gene expression profile?”* This query does not specify the type of cancer, it does, on the other hand, restrict all desired models to those based on gene expression data. This query may be posed by a researcher in pharmacogenomics looking to correlate the expression of specific genes with the biological function of specific drugs.
- **Example Query 3:** *“What papers have compared multiple supervised learning methods for the prediction of cancer diagnosis based on gene expression data using a cross validation method?”* This query could be posed by a clinical researcher in possession of a gene expression dataset who is looking for proven methods to build and validate models for diagnosing prospective cancer patients using gene expression microarrays. Notice that in this query, the specific disease and the specific outcome are not specified. Only the type of outcome is specified as “diagnosis”. Also notice that this query specifies classes of algorithms (“supervised learning”) and validation methods (“cross-validation”) rather than individual methods.
- **Example Query 4:** *“What datasets originating from breast tumor samples contain mass spectrometry data and contain clinical survival data?”* This is a specific query by someone who is interested in building and testing models that predict survival in breast cancer based on raw mass spectrometry data.

These queries require the search and retrieval of a multiplicity of molecular medicine modality object types including but not limited to documents, which are the focus of traditional information retrieval problems. Our envisioned system is intended to represent and retrieve four different types of objects relevant to clinical bioinformatics:

- **Papers:** A published paper is the primary unit of scientific communication. Individual papers or groups of papers describe the methods and results of high throughput molecular medicine research.
- **Datasets:** In many cases, researchers publish their data in the public domain (Broad Institute 2005). Often, that data is utilized by other researchers seeking to develop new and improved analysis methods, to test novel hypotheses, or simply to reproduce or validate the published results.
- **Algorithms/Software:** Research laboratories that develop data analysis methods often publish implementation of the algorithms that they have developed and applied (Broad Institute 2008).
- **Models:** Predictive computational models are produced by the application of algorithms on research datasets. Predictive computational models provide a “decision” based on molecular assays and clinical data obtained from a single patient. The predictive computational model’s decision (output) may then be used for the clinical management of the respective patient, for example to help determine the choice of effective therapy. Ideally the process of decision model formation includes rigorous statistical validation to ensure that the utility of a given decision model can generalize to a wider population.

## Related Work

Existing information retrieval systems specialized for molecular medicine modalities store and organize only related *subsets* of clinical bioinformatics research information. For example, PharmGKB (Altman et al. 2003; Oliver et al. 2002) is a database that links genomic variability, mostly accounted for by single nucleotide polymorphisms (SNPs), with phenotypes relating to pharmacokinetics, pharmacodynamics, or therapeutic clinical outcomes. Information is organized in PharmGKB by gene, drug, disease, publications, or datasets. ONCOMINE (Rhodes et al. 2004; Rhodes et al. 2007), a database and web-based analysis and visualization tools, is restricted to cancer-related gene expression microarray experimental results. Datasets in Oncomine are profiled (annotated) by cancer and tissue types, by experimental methods, and by the types of gene expression differential analysis performed on these datasets, e.g. comparing gene expression differentials across different prognosis groups or across different histological subtypes. Oncomine provides links to the original datasets as well as analysis tools for (clinical) differential analysis of these datasets, but does not store or classify the applied algorithms or inferred models that were reported in the original publications. The Gene Expression Omnibus (GEO) (Barrett et al. 2007; Edgar, Domrachev, and Lash, 2002), is a resource developed by the NCBI as a MeSH-indexed public repository of microarray and other forms of high-throughput “omics” data submitted by the scientific community. Sources of data in GEO include gene expression microarrays, ArrayCGH, SNP Arrays, Serial Analysis of Gene Expression (SAGE), Massively Parallel Signature Sequencing (MPSS), protein arrays, and mass spectrometry. Information in GEO is organized by series (study-centered data) or by individual genes. Many journals require that gene expression results be submitted in MIAME-compliant format (Brazma et al. 2001) to the GEO prior to publication (Ball et al. 2004). Some of the series in GEO are further curated and stored as datasets with more structured annotations (relevant citations, organisms) and the possibility to perform online data analysis. The Biometric Research Branch at the NCI has developed array analysis tools for gene expression data, and provides a hand-curated archive of human cancer gene expression datasets (Simon and Zhao, 2008). The Rembrandt (National Cancer Institute, 2005) repository is highly annotated for clinically-oriented outcomes but is restricted to brain-cancer-related molecular research data.

In addition to the above, formalisms and tools have been developed to allow genomic and proteomic researchers to ask questions of diverse data repositories. Such cross-database information queries benefit from standard and controlled representation of domain knowledge (Aitken, Webber and Bard, 2004; Smith et al. 2005). By standardizing and controlling domain concepts, ontologies such as the NCI Thesaurus (Sioutos et al. 2007), the Gene Ontology (GO) (Ashburner et al. 2000) and the Clinical Bioinformatics Ontology (REFSEQ) (Hoffman, Arnoldi and Chuang, 2005) support interoperability between clinical bioinformatics repositories. Ontology-based frameworks, such as the RAD/RAPAD Study Annotator (Manduchi et al. 2004), the Functional Genomics Experiment Model (Jones et al. 2004; Jones et al. 2006), and the Ontofusion system for biomedical database integration (onso-Calvo et al. 2007; Perez-Rey et al. 2006), support cross-database queries. Description logic(DL)-based languages (Baader, 2003), such as the Web Ontology Language (OWL) (McGuinness and van Harmelen, 2004) are popular means of formal ontology representation. DLs can be used for conceptual modeling, information integration, and support for semantic query mechanisms. As such, none of these resources provide a general-purpose information retrieval framework for clinical bioinformatics predictive models and related modalities as befits our goal.

## Model Formulation and Proof of Concept

### Model: Objects, indexing scheme, and queries

We developed an information retrieval model to support our intended system by examining use cases that mimic the queries introduced above in the domains of diffuse large B-cell lymphoma (DLBCL) and breast cancer. The model is described in the context of the task of retrieving research information from the semantically complex clinical bioinformatics domain of gene expression microarrays in the diagnosis and treatment of DLBCL.

Initially, we conducted manual literature reviews for papers that describe this domain. We noted the different objects that were described in the papers that were reviewed, i.e. by identifying *Algorithms*, *Datasets*, or *Models* described in each *Paper*. Conceptually, the objects in the knowledgebase are all the *Papers*, and the union of all *Algorithms*, *Datasets*, and *Models* that are described by the *Papers*. An *Algorithm*, a *Dataset*, or a *Model* can be referenced in more than one *Paper*.

Further examination of these objects revealed that each can be described by at least one Context that specifies the following elements in a tuple: <Disease, Population, Purpose, and Modality>. For example in the *Paper* by Wright et al. (Wright et al. 2003), a *Model* that predicts the molecular subtype of DLBCL was produced and validated by applying the *Algorithm* “Bayes Classifier” on two gene expression *Datasets*. The five objects (1 *Paper*, 1 *Algorithm*, 2 *Datasets*, and 1 *Model*) can each be annotated with the following *Context*: (*Disease* = DLBCL, *Population* = Human Patients, *Purpose* = Predict Molecular Subtype, *Modality* = Gene Expression Microarray).

**Figure 1:**
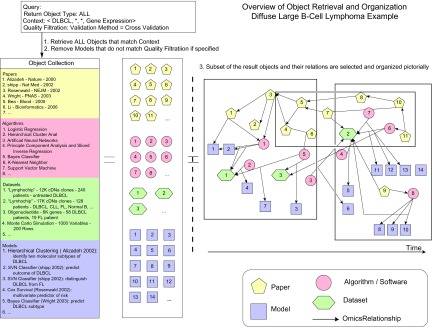
An overview of how the information retrieval model will be applied to the DLBCL use case. **Left side:** After specifying the desired query parameters (Context, Quality Filtration), the system will return a potentially large result set of molecular medicine modality objects. This enumerated set of objects is the raw result. Please refer to the subsection “Model: Objects, Indexing Scheme and Queries,” last two paragraphs. **Right side:** One or more subsets of the raw result may then be selected by the user for visualization and organization based on the relationships between these objects. The subsection “Model: Object Relationships and Quality Filters” elaborates on this process. The full details of the DLBCL use case are mentioned in the subsection “Proof of Concept: Diffuse Large B C-Cell Lymphoma”. Three subsets of objects from the DLBCL domain along with their relationships are organized pictorially according to our model in Figures 2, 3 and 4.

A query to the knowledgebase should then return a subset of the objects in the knowledgebase. A simple enumeration of *Papers, Algorithms, Datasets,* and *Models* that relate to gene expression microarrays in the context of DLBCL is shown in the left side of Figure 1. We also realized that a query can be represented as a partial or complete *Context*. For example, the *Contexts* represented by the example queries above are shown in Table 1. Queries 1–3 specify partial *Contexts*, and Query 4 specifies a complete *Context*. A quick and simple indexing scheme can be achieved by using a set of canonical terms for each of the *Context* elements, and then indexing each of the objects with at least one complete *Context* tuple. Objects are retrieved when their *Context* elements match the *Context* elements specified in the query.

**Figure 2:**
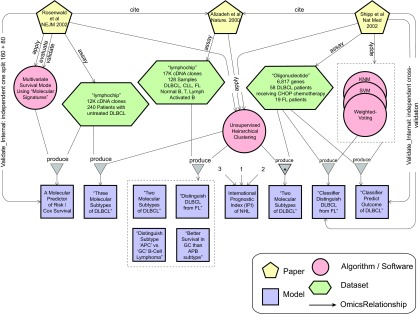
A pictorial representation of the first three widely cited *Papers* relevant to the DLBCL use case along with the *Datasets, Algorithms,* and *Models* that were described in these *Papers*. Identifying and presenting relationships between these objects is important for the semantic organization of this domain. These relationships are represented by edges connecting the different objects. For example, the three *Papers* each describe how *Algorithms* were applied to *Datasets* to produce decision *Models.* We identify this class of ternary relationship as *Run_ on_Produce* (*Produce* in the figure for simplification). Furthermore, the Shipp (Shipp and others, 2002) and the Rosenwald (Rosenwald and others, 2002) *Papers* describe how the rightmost and leftmost predictive *Models* (respectively) were validated using the *Datasets* that they had assayed. This scenario is detailed in the subsection “Proof of Concept: Diffuse Large B-cell Lymphoma,” paragraphs 1–3.

**Figure 3:**
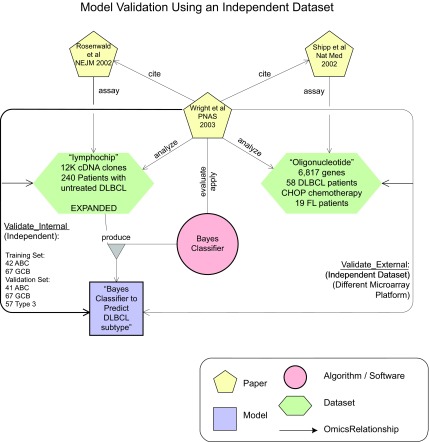
This figure shows the objects and relationships that surround the production and external validation of a Bayes-classifier *Model* as described in the Wright et al. (Wright and others 2003) *Paper* and explained in the subsection “Proof of Concept: Diffuse Large B-Cell Lymphoma”, paragraph 4. The *Model* (bottom center) was produced by applying the Bayes-classifier *Algorithm* to the lymphochip *Dataset* (left). The *Model* was internally validated (left side arc) using that Dataset which was split into independent training and testing sets. It was then externally validated (right side arc) using another independent *Dataset* that was assayed and described in a previous *Paper* (right). It is important to represent and identify this type of scenario in which higher quality *Models* are produced, i.e. *Models* that generalize across different *Datasets* and, in this case, across different molecular assay platforms (oligonucleotide vs. cDNA).

**Figure 4:**
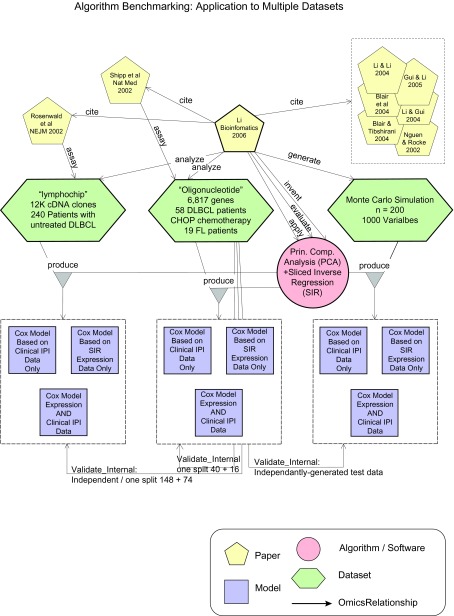
This figure describes how an *Algorithm* (PCA + SIR) was described by the Li et al. (Li, 2006) *Paper*. This *Algorithm* was benchmarked using two independent *Datasets* that were assayed and described by previous *Papers*, and one *Dataset* produced by Monte Carlo simulation. The *Models* that were produced by the application of this *Algorithm* on these *Datasets* were validated internally using one independent split of the respective *Datasets*. This scenario is commonly encountered in methodological research aimed at developing and benchmarking new classification *Algorithms.* Please refer to subsection “Proof of Concept: Diffuse Large B-Cell Lymphoma,” paragraph 5.

We conducted a broad search for DLBCL gene-expression-related objects, by placing a query as in Figure 1 that specified the following *Context:* (*Disease* = DLBCL, *Modality* = Genomic). In the following section we will discuss three clinical bioinformatics scenarios that involve a subset of DLBCL gene-expression-related objects. The scenarios were encountered when we analyzed the set of manually collected objects that satisfied this *Context*. Figures 2–4 will provide a pictorial representation of these scenarios.

### Proof of concept: Diffuse large B-cell lymphoma

DLBCL is the most common form of non-hodgkins lymphoma in adults. Historically, less than half of DLBCL patients are cured by chemotherapy (Vose, 1998). It was suggested early on that DLBCL actually comprises several diseases that differ in responsiveness to chemotherapy. A pioneering paper by Alizadeh et al. in 2000 (Alizadeh et al. 2000) applied bioinformatics methods to investigate this hypothesis. They measured gene expression levels in lymphoid tissue collected from a variety of healthy and sick individuals. The microarray platform used, called “lymphochip,” measured mRNA levels by hybridization on cDNA spots. The cDNA gene library on the lymphochip was deliberately designed to include genes known to be expressed in lymphoid tissue. The resultant *Dataset*, which consisted of around 17 thousand gene expression analytes for 128 samples, was analyzed using an unsupervised hierarchical clustering *Algorithm*. Based on the hierarchical clustering results, multiple decision *Models* were generated that either related to the biological behavior of DLBCL or to the clinical outcome of patients suffering from DLBCL (See Fig. 2). In the former category, the decision *Models* seemed consistent with the following hypotheses: (1) That DLBCL can be distinguished based on gene expression data from follicular lymphoma (FL), another form of lymphoma; (2) That there are two molecular subtypes of DLBCL; and (3) That one subtype’s molecular signature resembles that of activated peripheral B-cells (APB-like) whereas the other’s signature resembles that of B-cells found in the germinal centers of lymph nodes (GC-like). The resultant clinical decision *Model* of this study was that DLBCL samples that clustered in the GC-like category had better survival than those that clustered in the APC-like category.

Two subsequent studies attempted to further investigate and validate the hypotheses that were reported in the Alizadeh *Paper*. See Figure 2 for a graphical view of the objects and relationships that were reported in these three *Papers*. Rosenwald et al. used the same microarray platform, the lymphochip, to collect data from 240 patients with DLBCL (Rosenwald et al. 2002). In this study, two *Algorithms* were used. An unsupervised hierarchical clustering *Algorithm* was used in a similar way to that described in the Alizadeh paper. However, three resultant hierarchical clusters (molecular subtypes) were found and labeled: “Activated B-Cell-like”, “GC-B-Cell-like”, and “Type 3”. The second *Algorithm* relied on multivariate regression techniques to construct a clinical survival prediction *Model* based on (so-called) gene expression scores. The decision *Model* was derived from a *Dataset* of 160 patients and was validated on the remaining 80 patients. This decision *Model* instance was compared to another widely used clinical predictive *Model*, the “International Prognostic Index” (IPI) (The International Non-Hodgkin’s Lymphoma Prognostic Factors Project 1993), that predicts clinical outcome based only on clinical parameters. Molecular and clinical data were reported as independent factors in predicting clinical outcomes.

In a third study, by Shipp et al. (Shipp et al. 2002), gene expression was measured in tumor samples from 58 DLBCL patients receiving the CHOP chemotherapy protocol, and from 19 FL patients. In this study, however, oligonucleotide-based microarrays were used instead of the cDNA-based lymphochip. Supervised learning methods (*Algorithms*) were used to construct two predictive classifiers (decision *Models*): one associated with the biological hypothesis that DLBCL can be distinguished from FLbased on gene expression data, and another associated with the clinical hypothesis that gene expression data can predict the clinical outcome of DLBCL. The latter decision *Model* was also compared to the IPI clinical predictive *Model*, and in this study as well, molecular and clinical data were found to be independent factors in predicting outcomes. A more rigorous cross validation method was used to validate the models produced by this study. In this paper, the previous claims about molecular sub-types were put to test. The same unsupervised hierarchical clustering *Algorithm* was applied on their dataset^1^ to cluster the samples. Two molecular subtypes did emerge, and they did show “APB-” and “GC-” B-cell-like expression patterns. However, survival was *not* found to be different between the two groups.

Wright et al. (Wright and others 2003) wanted to reconcile the results from the last two studies (See Fig. 3). They developed a Bayes classifier (i.e. a decision *Model*) to predict molecular sub-type and clinical outcome. It was trained and validated on the Rosenwald *Dataset* that used the lymphochip platform. The classifier was then independently validated on the *Dataset* produced by the Shipp group, again using sequence annotations to reconcile the cDNA sequences with the oligonucleotide sequences. This seems to support the biological hypothesis that the “two molecular subtypes” in DLBCL correlate with different biological and clinical behavior. The semantics of the relationship between this *Model* and these two *Datasets* is reflected through the visual description and organization in this figure.

On the other hand, the more recent paper by Li et al. (Li, 2006) describes a study that develops and evaluates a specific data-analysis method (i.e. *Algorithm*) (See Fig. 4). This *Algorithm*, “Principle Component Analysis and Sliced Inverse Regression”, was applied to both the Rosenwald and Shipp *Datasets*, as well as to a *Dataset* produced by a Monte Carlo Simulation. Decision *Models* were generated and they were validated on an independent subset obtained through one split of the data (148 training samples, 74 training samples). This figure focuses on one algorithm in this *Context* and relates all the objects (and relationships) that are relevant to the evaluation of this *Algorithm*.

### Model: Object relationships and quality filters

These examples demonstrate that the figures and their underlying complex semantics can not be conveyed by simple retrieval and enumeration of objects returned by *Context,* i.e. as in the left side of Figure 1. A potentially large number of returned objects need to be organized and displayed intuitively. One aspect of object organization relates to the relationships between the different object types. Such relationships were indicated by edges in the figures. For example, a *Paper* can describe how an *Algorithm* is used to *Analyze* a *Dataset*. A *Model* is *Produced* by running an *Algorithm* on a *Dataset. Models* are *Validated* using more than one *Dataset*. Grouping objects in annotated relationships can be leveraged in post-retrieval organization and display to provide semantic information about the objects.

All the predictive *Models* mentioned above underwent some form of validation, expressed via the *Validate* relationships in the respective figures. The *Validate* relationship is further specialized via the *Validate External* and *Validate Internal* subclasses. Please see the section on evidence annotation in the appendix. As molecular predictive *Models* mature and get closer to routine clinical practice, it is important to consider the evidence supporting their validity and generalizability. As described by Pepe et al. (Pepe et al. 2001), clinical bioinformatics predictive models typically go through multiple stages of validation before being accepted in standard practice. Therefore, our envisioned system will need to filter different objects based on the strength of supporting evidence. For example, these query results can be narrowed to include only high quality models by appending the following requirements to the query *“[get models that …], have been developed using datasets with sample size (n) larger than 200 patients, and that have been validated using an independent dataset.”*

The concepts mentioned so far that will support the information retrieval model are described in more detail in the appendix. Now we can revisit Figure 1 in its entirety. It gives an overview of how a query is intended to be processed: A query sets the desired object types, specifies a partial or complete *Context*(s), and sets conditions for quality filtration. The process is decomposed into three steps: (1) returning objects that are indexed by *Context* tuples that match the query’s *Context*, (2) filtering out objects based on quality of evidence, and (3) selecting smaller sets of objects by the user and organization of these objects along with their relationships in an intuitive way.

### Proof of concept: Molecular prognostic test for breast cancer—MammaPrint^®^

**Figure 5:**
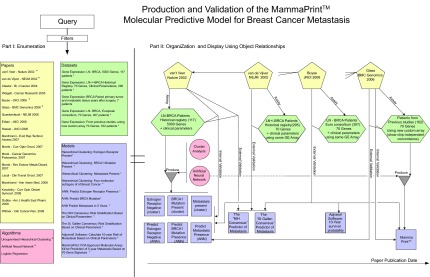
This figure depicts objects and object relationships that span the development and evolution of the MammaPrint^™^ *Model* from its earlier versions. The figure also represents the validation of MammaPrint^™^ across multiple *Datasets* and its comparison to other *Models.* Notice that the other clinical predictive models are classical models that do not incorporate molecular data. The information retrieval framework will incorporate classical (non-molecular) clinical predictive *Models* only when they are relevant to the validation of molecular prediction *Models*. Otherwise classical *Models* will not be indexed or stored. Similar to the process described in Figure 1, a query to this domain will return a raw set of objects (Part I, left side). A subset of the raw result may be selected for visual organization and display (right side) of the objects and their relationships (Part II, right side). The detailed prose description of this scenario is presented in the subsection “Proof of Concept: Molecular Prognostic Test for Breast Cancer—MammaPrint^®^”.

The same semantic representation and organizational principles of *Papers, Datasets, Algorithms,* and *Models* that relate to MammaPrint^®^, the first commercial Breast Cancer molecular prognostic test, are shown in Figure 5 and explained below.

Researchers in the Netherlands (van’t Veer and others 2002) analyzed historical breast cancer tissues using a 25,000 sequence oligonucleotide microarray. Seventy genes were found to be predictive of 5-year metastasis in Lymph Node (LN)-negative female patients under the age 55. Unsupervised hierarchical clustering (*Algorithm*) distinguished the following three characteristics: Estrogen-receptor negative (i.e. can not be treated with the drug Tamoxifen), having BRCA1 germline mutation, and metastasis within 5 years. In other words, three *Models* were *Produced* using the hierarchical clustering *Algorithm*. A supervised machine learning method, Artificial Neural Network (ANN, another *Algorithm*), was used to construct a classifier (*Model*), using a “70-gene signature”, that predicts these characteristics. This predictive *Model* was *Validated Internally* using a leave-one-out approach. The researchers also showed that this molecular predictive *Model* was an independent predictor of metastasis from other well-known decision *Models* that relied solely on clinical parameters (the NIH Consensus and the St. Gallen Consensus). In that paper, not only did the molecular decision *Model* improve clinical outcome prediction, but it also predicted the same number of patients who had metastasis with fewer false positives. This is important given the morbidity and economic costs associated with adjuvant chemotherapy (Erban and Lau, 2006; Hassett et al. 2006). The 70-“gene signature” *Model* was *Externally Validated* (van de Vijver et al. 2002) using 295 consecutive historical patients in a *Dataset* that is different from the *Dataset* that was used to *Produce* that signature. It also provided (Weigelt et al. 2005) the correct decision outcome, i.e. *Externally Validated*, on primary tumor tissue from 7 patients and on matched metastatic tissue obtained years later from the same patients (not shown in Fig. 5). This validation was not of a clinical, but of a biological hypothesis that: molecular subtype determines the metastatic potential early in the disease as opposed to invasiveness resulting from cumulative mutations.^2^

A spin-off commercial company, Agendia^™^, developed a custom kit that measured gene expression and contained a similar 70-“gene signature” *Model*, now called MammaPrint^®^. MammaPrint^®^ was also *Produced* using the ANN *Algorithm* and *Internally Validated* (Glas et al. 2006). The new platform was shown to be concordant with the previous 25,000 oligonucleotide chip (Glas and others 2006) (thus *Externally Validating* that *Dataset’s* corresponding *Model*). MammaPrint^®^ was *Externally Validated* through multi-center European consortium study (Buyse et al. 2006). It was also compared to known clinical decision *Models*, including one based on a software, Adjuvant!, that calculates 10-year survival probability based on clinical parameters.

## Discussion and Future Work

Some public resources currently implement some but not all aspects of our intended functionality and not in an integrated retrieval framework as was discussed in this paper. For example, PharmGKB’s clinical outcomes are restricted to outcomes of therapy, and exclude diagnostic and prognostic markers. Oncomine’s representation and organization of oncology molecular datasets does not cover decision *Models*, the original *Algorithms* by which these models were produced, or their validation methods. *Datasets* and *Papers* are MeSH-indexed in GEO/PubMed, but their relationships to respective *Models*, *Algorithms*, and *Contexts* are not explicit. The proposed framework is designed to compliment existing resources and extend current representations to cover molecular clinical predictive models and their related modalities. Our choice to model this domain using an OWL ontology was made with the goal of semantic integration of this framework with existing knowledge sources. Whenever possible we associate objects in our database with their counterparts in external databases, e.g. using PubMed uid for papers and GEO accession numbers for datasets.

Most existing clinical predictive models do not incorporate molecular features. Classical predictive models that are purely based on clinical parameters are outside the scope of this information retrieval framework; however, classical models will be incorporated *only when* they exist within the context of molecular predictive models. For example, we did include the International Prognostic Index model in the DLBCL case study, and the St. Gallen Consensus model in the MammaPrint^™^ validation case study. Similarly, storing and annotating gene signatures that predict underlying biological behavior without clinical outcomes is outside the scope of this framework. Again, some molecular clinical predictive models incorporate aspects of purely biological signatures, so we will also include those *only when* they exist within the context of clinical models. For example, the early DLBCL models (Fig. 2) that identified the underlying biological behavior of DLBCL (as APB-like or GC-like) did correlate with clinical outcomes and therefore they were included in the framework. Using molecular signatures that measure (EGF-R) receptor activity for choice of treatment with tyrosine kinase inhibiting drugs is another example (not discussed in this paper) that comes to mind of what will be included in this framework.

The focus of the present paper is the underlying information retrieval model and not the system’s implementation and inference mechanisms which will be described elsewhere (please see Appendix). When developing the formalisms described in this paper, we deliberately selected the minimal set of classes and properties that is expressive enough to allow for semantic organization of the domain. This level of simplicity is intended to enable automated methods for building the knowledgebase. Our current research is focused on building and validating machine learning models that can correctly annotate the *Contexts* described in clinical bioinformatics papers, and that can also correctly identify the validation methods that are employed in those papers.

## Conclusion

While clinically-oriented research exploring gene expression microarrays, mass spectrometry, SNP arrays and other high-throughput molecular assays has followed an exponential growth in recent years, to date there is no general purpose system that allows researchers and clinicians to find models, papers, data, and other related information in this emerging field using a unified and friendly interface. In the present paper we propose a framework for such interface and demonstrate the complexity of its required functionality. Our long-term goal is to construct a system that addresses this need. As a significant first step, we developed a formalism that supports storage and retrieval of a multiplicity of clinical bioinformatics objects such as published papers, datasets, decision models, and discovery and inference algorithms. This formalism opens the way for automated methods that support the knowledgebase’s creation and annotation. In addition, it allows for a second layer of organization of objects returned by queries based on their (1) interrelationships and (2) strength of methodological validation. We demonstrated the power of this model in the complicated domain of diffuse large B-cell lymphoma. In future work we plan to deploy and test a prototype system based on the model of the present paper applied to biomarker discovery for other malignancies.

## Figures and Tables

**Figure 6:**
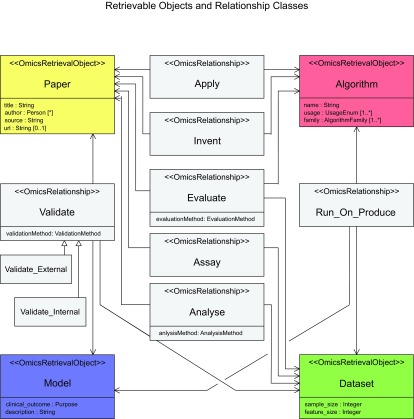
A UML diagram showing the four retrievable classes (subclasses of the abstract OmicsRetrievalObject class), some relationship classes (subclasses of the abstract OmicsRelationship class), and their associations. Some relevant properties of the retrievable classes are shown here as well. Apply, Invent, Assay, and Analyze are binary relationship classes, whereas the rest are ternary. The knowledgebase will contain instances of the retrieval and the relationship classes (as well as others not shown here, such as Context-related classes). For example, a given paper **p** (instance of *Paper*) may describe how a given model **m** (instance of *Model)* was validated using a dataset **d** (instance of *Dataset*). An instance **v** of the *Validate* relationship will be created referencing the objects **p**, **m**, and **d**. If **d** was the same dataset that was used to produce **m**, then **v** will belong to the *Validate_Internal*. class. *Validate_Internal* and *Validate_External* are subclasses of the ternary relationship, *Validate*. As such, they inherit its properties but offer more specialized properties such as specifying whether the validation method described by the *Validate_Internal* instance was done on independent samples within the related *Dataset* or not.

**Table 1:** Contexts partially or completely specified by the example queries in the problem statement section above

Query #	Disease	Population	Purpose	Modality
1	DLBCL	Human Patients	Response to CHOP Regimen	-
2	-	-	Response to CHOP Regimen	Gene Expression
3	-	-	Diagnosis	Gene Expression
4	Breast Cancer	Human Patients	Predict Survival	Mass Spectrometry

## Footnotes

1 Notice that the oligonucleotide sequences on the microarrays platform of this study were matched through their annotations to the cDNA genes in the “lymphochip” platform used in the other studies. Only the sequences that matched were used in this clustering technique. That’s why the ternary relationship apply-on-to-produce has an asterisk in Figure 2.

2 That same study *Validated* a decision Model described elsewhere (also not shown in Fig. 5) that used unsupervised clustering to separate Breast Cancer samples into four molecular subtypes. All matched primary tumors and metastatic tissue belonged to the same molecular subtype.

## Appendix

### Context indexing and automation

As mentioned earlier, an object’s *Context* is represented by a tuple that specifies *Disease*, *Population*, *Purpose*, and *Modality*. Whenever an object is described in a *Paper* that object is indexed by the *Context* with which it is described in that *Paper*. An object, e.g. *Dataset*, can be indexed by many *Contexts* because more than one *Paper* can reference the same object and in multiple contexts. For example, a “neural network” Algorithm, can be described in the following *Context* in one *Paper* (<DLBCL, Human Patients, Prognosis with Treatment, Proteomics>) i.e. neural network predictive *Models* were developed to predict prognosis in DLBCL using proteomic data. It can then be described in a different *Context* in another *Paper*. A *Paper* can be indexed by all the *Contexts* that apply to the objects in that Paper; however, individual objects described in a *Paper* are not necessarily described by all the *Contexts* that are mentioned in that Paper. For example, a *Paper* that evaluates a certain *Algorithm* using multiple *Datasets* drawn from multiple diseases can be indexed by *Context* tuples that reflect all the diseases, but each individual *Dataset* can only be indexed using tuples that reflects its specific disease.

We use a canonical set of terms to specify the individual elements of a *Context* tuple. Initially we are only covering Neoplasms, and we will adopt the following nomenclature for *Disease*: Breast Neoplasms, Lung Neoplasms, Colorectal Neoplasms, Prostatic Neoplasms, and so on to cover all neoplasms in the domain of clinical bioinformatics. *Population* refers to one of three types: Human Patients (Datasets created by assays on tissues taken from patients, this can include normal tissue taken as control), Cancer Cell Line, and Animal Model. *Purpose* refers to the type of clinical outcome, we have determined four categories of clinical outcomes: (1) Diagnosis, i.e. using a computational *Model* to assign a diagnostic label based on molecular profile, an example in this category is the well known AML/ALL classification *Dataset* by Golub et al. (Golub et al. 1999); (2) Prognosis with no treatment, (3) Prognosis with one treatment arm, e.g. 5 year survival or metastasis prediction for patients on standard treatment; and (4) Prognosis with more than one treatment arm. The latter refers to situations where molecular computational models predict whether patients benefit from certain treatments, e.g. hormone therapy susceptibility based on molecular pathway activations. It also includes situations where the biological effect of certain chemicals, e.g. when tested on cancer cell lines, is measured. Finally, we determined three categories for *Modality*: (1) Genetic, refers to high throughput modalities that assess inherited genetic characteristics, e.g. SNPs and haplotypes; (2) Genomic, refers to high throughput modalities that assess functional genomic characteristics of disease or disease-related tissues, e.g. gene expression microarrays, array CGH; and (3) Proteomic, e.g. high throughput modalities like Mass Spectrometry and Gel Proteomics.

There are a plethora of reference ontologies (Burgun, 2006) and other formalisms that can represent *Context* elements with high granularity, e.g. SNOMED-CT for Disease and Purpose. A very expressive annotation of *Context* elements using complex ontologies with extensive subsumption hierarchies has many benefits. However it is labor intensive and with current and foreseeable technology relies heavily on human operators. As explained, our aim is to accelerate the indexing and annotation of *Papers* using automated or semi automated means.

### Classes, Objects and relationships

We chose to represent the different object types, their relationships, as well as other entities in the clinical bioinformatics domain using Description Logic. Using Protégé’s OWL plug-in (Knublauch, Musen and Rector, 2004), we developed an ontology (Discovery Systems Laboratory, 2008) that uses OWL axioms to define classes (concepts) of clinical bioinformatics entities and their respective properties (attributes). We chose OWL because the supporting tools are readily available, because we can use it to represent the domain unambiguously, and because we can use it to share our representation. We note that our aim is *not* to build extensive DL-based knowledgebases *or* to develop reference ontologies.

The main classes are *Papers*, *Datasets*, *Algorithms*, and *Models. Datasets* can have simple properties such as dataset dimensionality and sample size or complex ones such as related diseases and population characteristic. *Algorithms* are annotated with properties to reflect the different methodologies e.g. “supervised” vs. “unsupervised learning”. Decision *Models* are annotated by the specific outcomes that they predict.

The semantics of relationships between classes in clinical bioinformatics is captured through *relationship classes*. For example, a *Paper* “proposes” or “invents” a specific *Algorithm*, “evaluates” that *Algorithm* using a *Dataset*, or simply “applies” that *Algorithm* on a given *Dataset*. So in addition to *classes of objects*, the ontology specifies *classes of relationships* between classes. Most relationships are binary, although there are some that are of higher arity. Relationships in our ontology are represented as classes and not properties (or “roles” in DL jargon). Our reasons for that include: (1) uniformity in representing all relationships, a significant fraction of which is not binary and thus cannot be represented by a DL-role, and (2) the need for rich annotation of the relationships themselves. For example, the relationship *Validate_Internal* (when a model is validated within a study) requires further annotations such as the type of validation performed (independent prospective sample? N-fold cross validation? Leave One Out cross validation?) Modeling relationships using classes instead of roles will add complexity to reasoning; however, for the foreseeable applications, we envision that a relational database with indexed relationship tuple tables will be adequate (for implementation and reasoning) for typical queries. Please see section on inference and implementation. Using classes to model relationships may also make reuse of this ontology more cumbersome, and is a limitation of this ontology. The four retrievable classes along with a subset of relationship classes are shown in Figure 6.

Research and discovery within the domain of clinical bioinformatics can be conceptualized as an overarching process that consists of: (a) collection of high-throughput molecular profiling data through molecular assays, (b) analysis of such data using specialized techniques, and (c) generation and validation of respective decision *Models*. These processes can be represented via a set of axioms that constrain relationships between classes in our ontology. Such constraints represent implicit domain knowledge such as: “In a *Paper*, one or more *Datasets* are assayed,” or “An *Algorithm* is applied on a *Dataset* to produce a *Model*”. Some of those constraints can be inferred from the UML diagram in Figure 6.

Currently, relationships between objects are manually annotated. Annotated relationships will be used to support the third step in the query process (semantic organization and display). These relationship instances are indexed and will be used to construct edges between the objects returned by the query and to drive the visual organization of results.

### Support for evidence annotation and filtering

As mentioned earlier, decision *Models* vary in the degree of validity and of generalizability outside of the population from which they were formulated. This variability results from the different methods with which the investigators validate their models and from the different experimental designs.

The performance of decision *Models* is usually evaluated on independent samples within the study *Dataset*, or on *Datasets* collected from different studies altogether. The former case is represented through the *“Validate_Internal”* relationship, and the latter through the *“Validate_ External”* relationship. Both are subclasses of the *Validate* ternary relationship class (Fig. 6). Note that internal validations are sometimes done on non-independent samples. This is a bad practice that likely leads to over-fitting of the resultant decision *Models*, and is therefore an important attribute to highlight when displaying results. The *Validate_Internal* relationship is annotated as being done on either non-independent or independent samples.

The class *ValidationMethod* is a property of the *Validate* relationship class. Instances of this class correspond to specific validation methods such “Leave-One-Out Cross Validation,” “N-Fold Cross Validation,” etc. Statistical (Aphinyanaphongs et al. 2005; Wilczynski et al. 2005) classification methods have been used successfully before to classify the nature of evidence based on document content. We plan to automatically identify the *ValidationMethod* classes based on *Paper* contents.

### Brief discussion of inference and implementation

This paper addresses representational requirements of the information retrieval task at hand and the expressiveness of the model and underlying formalism. However we will briefly discuss inference and implementation of this model. In the first phase of our work, the papers were collected and organized manually. As we added more objects, and as the model was formulated we found that a simple relational model was enough to store and execute our simple queries. The objects were stored in their own tables, the relationships between the objects were stored in join tables, “Context” tuples were stored in a separate table, etc. It can be easily shown that matching the pattern of a “Context” query can be done via simple SQL queries that are dynamically generated. With the correct choice of index keys, the retrieval process has been very efficient and we expect it to scale efficiently for simple queries. We used a simple (PHP-based) web framework with a browser interface and a MySQL database backend to build an application for storing and retrieving representations of our objects and their relationships. We have not yet implemented graph extraction and visualization. Graph extraction should be a trivial problem (identifying objects a certain depth from a model of interest, filtering out/in objects with specific properties, etc.) Graph visualization can be done via any of available graph-layout software (e.g. Graphviz). Graph elements can be passed to a web browser for rendering using a mark-up standard like SVG.

Semantically, we modeled the relevant objects of the domain, their relationships and the domain knowledge using OWL-DL axioms. This OWL file is available for download as indicated earlier. This leaves the door open for future storage and retrieval of the objects using DL-based databases and query languages; however, we do not see a need in the near future for DL-based inference and implementation. We think that using OWL to model the domain will facilitate semantic integration of this framework with other resources in the future. We envision implementing this framework as a web service that will be compatible with standard web services technology.

The inference task that we find most challenging is the automated identification of relevant papers from the literature and the automated annotation of the objects (for now only papers) by the correct “Context” tuples. Again, using automated or semiautomated methods is essential for building a comprehensive and up-to-date knowledgebase. This has motivated our drive towards simple representation formalism. Our current work is focused on building machine learning filters for identifying and annotating domain papers using text categorization, and on investigating different approaches for tuple extraction. The purpose, and subsequent evaluation, of this effort is done along two lines. The evaluation of information retrieval recall and precision is done using a human-annotated corpus of papers that serves as a gold standard (currently exists for two domains, Lung Cancer and Breast Cancer with more annotations by domain experts underway). The individual papers are labeled for many things such as whether they describe the domain of clinical bioinformatics, whether they correspond to single gene vs. high throughput experiments, as well as all the Context tuple assignments that apply to each specific paper. The second dimension of evaluation relates to the adequacy of these automated techniques as means for building the knowledgebase required for this purpose, and how users interact with the resultant system.
